# Super Water-Repellent Cellulose Acetate Mats

**DOI:** 10.1038/s41598-018-30693-2

**Published:** 2018-08-20

**Authors:** Fateh Mikaeili, Pelagia I. Gouma

**Affiliations:** 1 The Ohio State University, Department of Materials Science and Engineering, Columbus, USA; 2 The Ohio State University, Department of Mechanical and Aerospace Engineering, Columbus, USA

## Abstract

A single-step synthesis of super-water-repellent oil sorbents based on cellulose acetate (CA) mats is reported in this paper. Key phenomenological mechanisms involving roughness and changes in chemistry are used to describe the change in hydrophobic behavior of the CA mats. Contact angle calculations followed by Cassie’s model apparent contact angle prediction have shown roughness alone is not capable of producing the super-hydrophobicity exhibited by as-spun mats. Fourier transform infrared spectroscopy of spin coated and electrospun mats shows a significant difference in the stretching of the hydroxyl bonds of the two materials. As it is this hydroxyl group which adds to the overall polarity of surface thus hydrophilicity of the material, we propose that the electrospinning process not only creates a rougher surface but also alters the chemistry of the electrospun cellulose acetate mats which ultimately gives rise to the reported hydrophobicity. Finally, due to their water repellent nature, and oleophilicity of the as-spun mats were tested as oil sorbent mats. The as-spun mats were capable of absorbing thirty times their weight in oil demonstrating their application for oil-water remediation.

## Introduction

For a material to be considered super hydrophobic, it must display an apparent water contact angle of more than 150° and low contact angle hysteresis^1^. To find materials which exhibit this, one does not need to look very hard; a number of excellent examples of super hydrophobic surfaces can be found by simply examining nature. Numerous plant’s leaves across the globe exhibit hydrophobicity^2,3^ the tarsus of a strider^4^, the palm of a gecko^5^ and even the shell of some desert beetles^6^ all make use of hydrophobicity. Biomimicry and other attempts to make non-natural super hydrophobic surfaces^7,8^ have concluded that surface roughness and surface energy are the key characteristics that affect the hydrophobicity of a material^9–12^. Many attempts have been made to develop super hydrophobic materials by manipulating these two characteristics^13^.

As it pertains to increasing surface roughness, one processing technique that has gained some attention is electrospinning due to its simplistic setup and versatility in producible fibers^14^. These studies show that roughened electrospun fibers were then coated and modified to decrease the surface energy to achieve a super hydrophobic surface. Examples of methods and materials used for coatings on a roughened electrospun surfaces are TEOS:DTMS through sol-gel^15^, CF4 coating through Plasma Treatment^16^, PPFEMA coating using Chemical Vapor Deposition (iCVD)^17^ and Layer by Layer (LBL) fluorination of the surface^18^ and so on. Since it is apparent that in the formation of a super hydrophobic material in the mentioned studies a distinct second step was required, the role of electrospinning was always considered to be solely increasing the surface roughness and the providing air traps that shift the hydrophobicity of a material.

Cellulose acetate (CA) is one of the polymers that was used to study the hydrophilic to hydrophobic transformation via electrospinning^14–16,18^. It is a modified natural polymer produced by acetylation of cellulose, one of the most abundant and low-cost renewable natural polymers, used in a broad field of application such as textile, membranes, and filters^19–21^. Having one hydroxyl group (depending on the degree of substitution) in each glucose module of its structure, makes the drop coated surface of CA to be polar and therefore, naturally hydrophilic. Using the two distinct routes mentioned earlier, a hydrophilic to hydrophobic transition in CA was reported before^15,16,18^.

In this study, super water repellant CA fibrous mats were developed using electrospinning in one step without further surface modification. The as-spun CA fibrous mats show an apparent water contract angle of 154 degrees. The effect of electrospinning on the hydrophobicity is studied distinctively by studying the change in roughness and chemistry of CA. The effect of electrospinning on the roughness (and the subsequent contact angle) of the surface have been predicted through Cassie’s model on the as-spun fibers and shown to be almost 30 degrees away from what was achieved in the lab, which proves the earlier assumption regarding the need of further surface modification to explain the super water repellency achieved after electrospinning. Fourier Transform Infrared spectroscopy (FTIR) of the as-spun and as received material showed a significant decrease in the stretching of the hydroxyl bonds after electrospinning. We propose that in the case of CA, electrospinning simultaneously roughens the surface and modifies the surface energy ultimately creating a super hydrophobic material in a single step. Due to its super hydrophobicity and environmental compatibility, the effectiveness of as-spun CA matts has been tested as a potential replacement for traditional oil spill remediation products.

## Experimental

CA (Sigma Aldrich, USA) with the molecular mass of Mn~30,000 was used in this experiment. The binary solvent system used to dissolve CA consisted of acetic acid (Sigma, ACS reagent grade) and acetone (Sigma, ACS reagent grade) with a volume ratio of 2/3. CA was dissolved in acetone/acetic at room temperature with a series of different concentrations through ultrasonic agitation for 2 hours. The solution was then kept untouched for 20 hours. Cast films of CA were also prepared on glass slides through spin coating (Chemat Technology, Spin Coater KW4) technique for evenly spreading the CA solution. The thin CA cast films were dried in air for 30 minutes. Electrospinning was carried out using a high voltage of 20 kV. The syringe pump was programmed to dispense the solution at a flow rate of 30 μl/min. The working distance was remained constant at 7 cm and a grounded Aluminum foils was used to collect the fibers. All fabricated materials were characterized by contact angle (CA) measurements (EasyDrop Model 100-00) using sessile drop technique with deionized water droplets. All reported contact angle data is obtained by averaging 3 points on each surface. For each one of the samples, 2 different surfaces were tested. Scanning electron microscopy (SEM) was used to observe the morphology of the nanofibers after electrospinning. Scanning electron microscopy (SEM) images of original cellulose fiber were obtained using a Hitachi S4800 FE SEM. In order to ensure conductivity and reducing charging effects on the surface, an appropriate size fiber sample was coated by Gold-Palladium sputtering. Fourier transform infrared (FTIR) spectroscopy was carried out using a ATR-FTIR spectrometer (Nicolet Magna 560 FTIR Spectrometer). Experiments were run in triplicate from two different surfaces and the results were normalized.

## Results and Discussion

**Figure 1:**
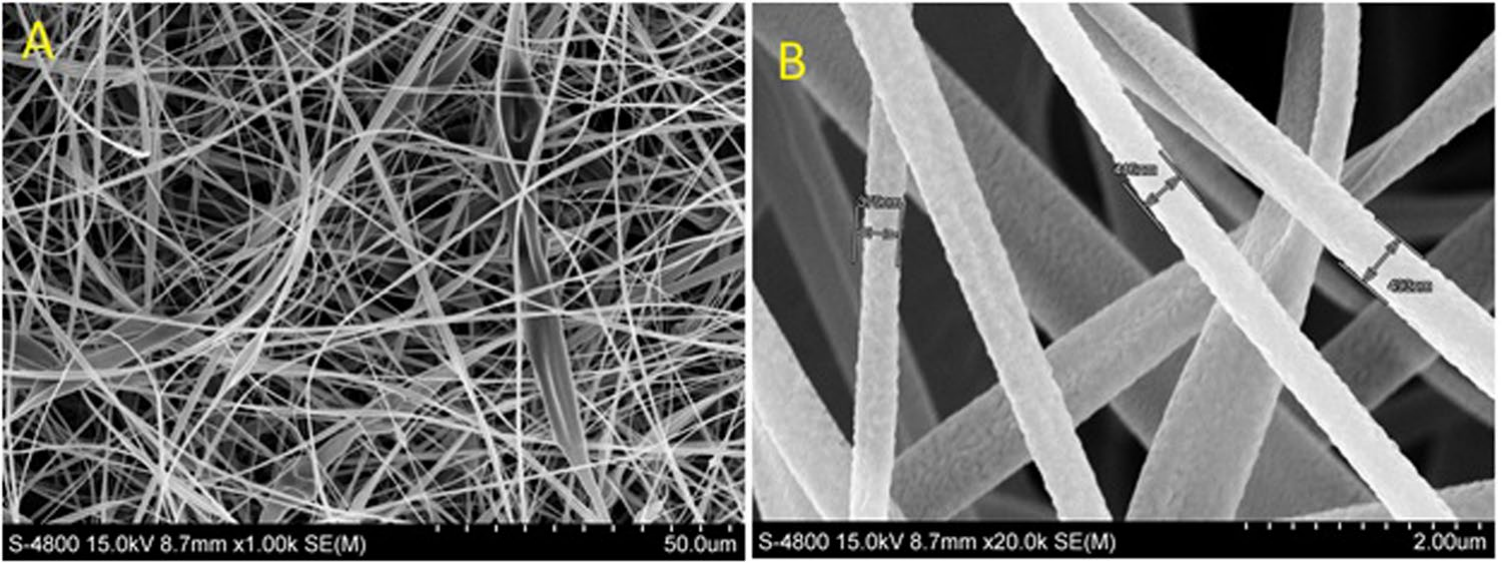
SEM Images of the as-spun fibers.

As-spun CA mats consisted of several layers of randomly oriented fibers 350–400 nm diameter with open porosity throughout which is shown in Fig. 1. The high magnification SEM micrographs showed some folded fibers without any beads.

**Figure 2:**
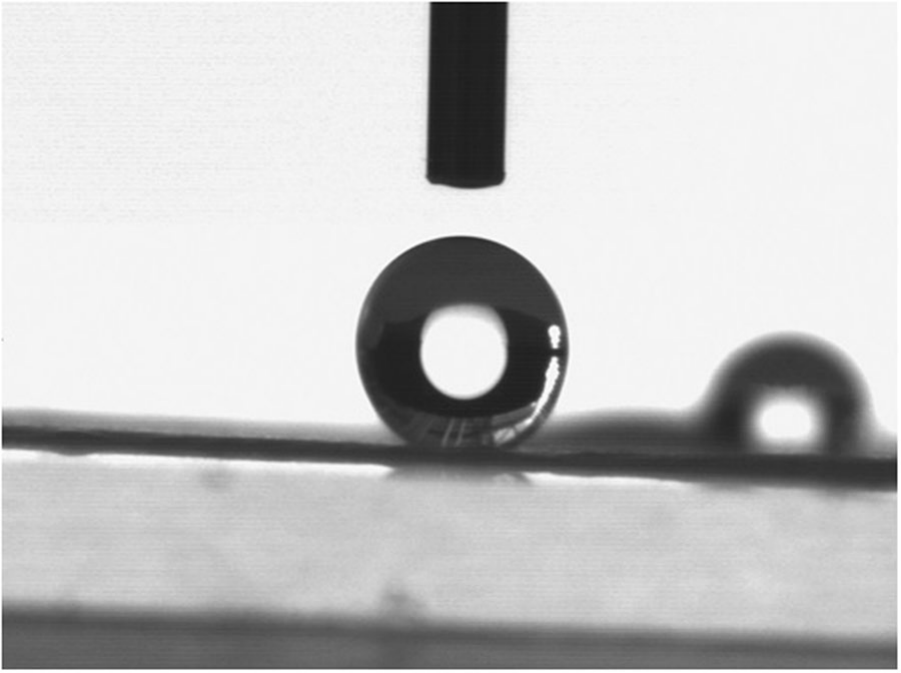
Water Contact angle of as-spun CA fiber with concentration of 15% w/v.

Figure 2 shows the results of a water contact angle test on the fibrous mat vs. a spin-coated thin film of the same material. It is generally agreed that a hydrophobic surface shows a high water contact angle (*θ* > 90°)^22^. The Nano fibrous mats with 15 percent w/v CA showed the highest contact angle that can be seen in Fig. 2. Measurement of water contact angle on spin-coated CA thin film reads 63.67, indicating the surface to be hydrophilic. Whereas the water droplet on CA fibrous mats, Fig. 2, gives a contact angle of 154.3 thus being super-hydrophobic in nature.

Since the static water contact angle may decrease with time due to various reasons such as evaporation of the water droplet or the surface alterations, water contact angle is usually recorded with respect to time. All of the water contact angle mesurements in this work were taken for 60 second and in 5 second intervals; no significant change was obsvered in any of the as-spun or as-cast samples. To take this test further, the buoyancy of the same Cellulose Acetate as-spun mats was preformed by our group in another work^23^, and it was reported that the these were stable, floatig on water without absorbing any water for 17 weeks.

**Figure 3:**
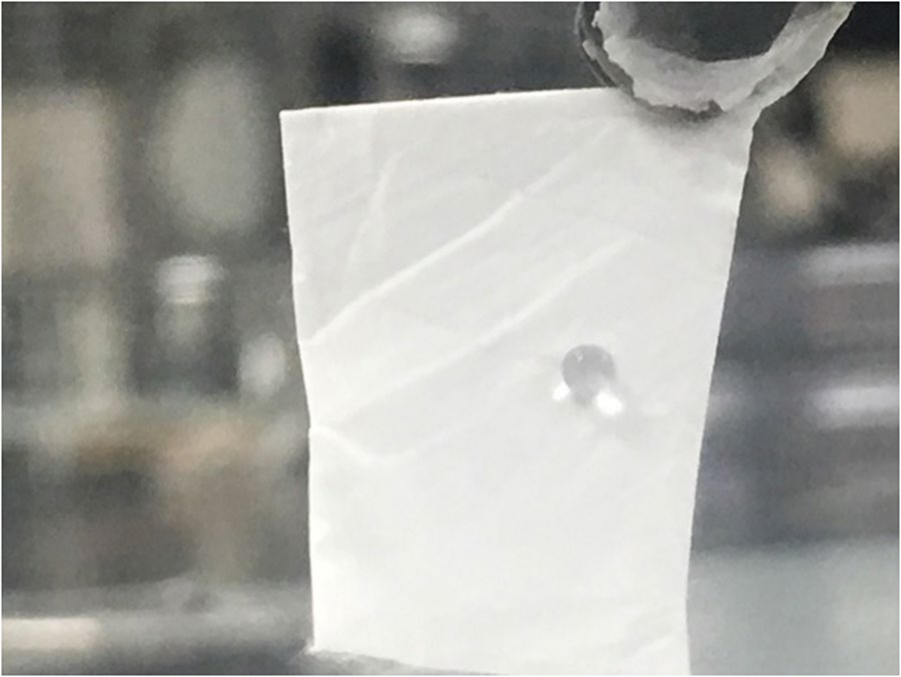
Demonstration of the roll of angle of a water droplet on electrospun CA fiber when the surface is tilted at 90 degrees.

Meanwhile, defining a super hydrophobic surface just by measuring contact angle is a matter of debate^24^, measuring the hysteresis of rolling angle is stated to be one of the measurements required to define a super hydrophobic surface when it is designed to be a self cleaning substrate. Calculating the hysteresis of the rolling angle of CA as-spun fibers exhibited a very high angle which makes this sample not suitable to be considered as a self-cleaning surface. Figure 3, shows that even when the as-spun fiber with a water droplet on it was tilted at 90°, the droplet did not start to fall off. Therefore, fabricated electrospun CA fibers is addressed as a super water repellant fibers.

The effect of roughness on water contact angle was developed in two independent studies by Cassie^9^ and Wenzel^12^. Assuming that water takes the shape of the surface and shadows all variations in surface morphology, Wenzel proposed that on a rough surface, Water-Solid interface is increased and then he derived an interdependent equation relating roughness and water contact angle. On the contrary, Cassie’s model suggests that a rough surface like lotus leaf is super hydrophobic because of the air packets trapped in the topological variations of the surface and suggested the following equation,

$$cos\,{\theta }_{c}=f\times cos\,{\theta }_{\gamma }+f-1$$

where, ${\theta }_{\gamma }$ is the contact angle of a smooth surface, f is the ratio of the solid in contact with liquid^25,26^.

Cassie’s theory assumes that the porous surface of CA fibrous mats may trap air between the bottom surface and water droplets resulting in high contact angle^27^ and a coating with low surface energy group like HF may decrease the surface energy of the mat to make a super hydrophobic material. This explanation was used in many of the previous studies on hydrophobic electrospun CA mats; however, to study if the very high contact angle of 153° achieved by electrospinning 15% w/v of CA is a result of only surface roughening, this model is applied in this study. By doing so, we can calculate the effect of roughness and air traps emerged during electrospinning on the contact angle of the mats.

By applying Cassie’s model [S1], we found that the maximum water contact angle with the roughness aquired after electrospinning can not exceed 122.2° since the least amount for the F factor in different concentrations is close to 0.3. In other words, the F factor of a completely flat surface with the contact angle of 63° should be lowered to at least 0.1, in order to achieve super-hydrophobic contact angle region. This number is far from what was achieved after electrospinning, proving the present assumption in the litreature that roughened surface through electrospinning alone, is not enough. Also proving that in the case of experiment explained in this paper, since the high contact angle was achived without further modification, there should be another reason, mainly changing the surface energy of the mats.

**Figure 4:**
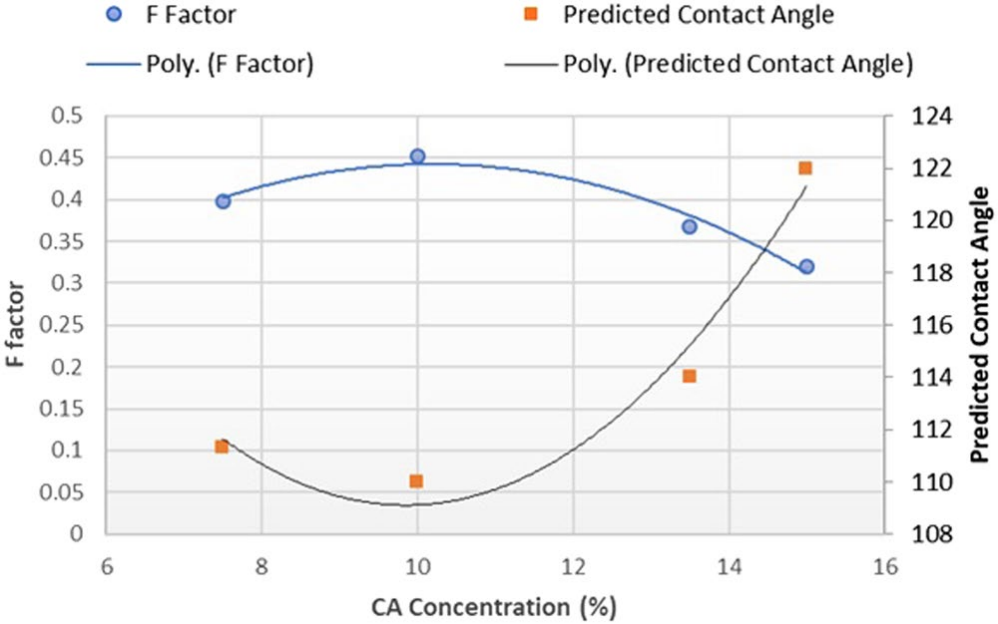
Dependence of F factor and Predicted contact angle to CA concentration.

In Fig. 4, dependence of CA concetration on F factor in Cassie’s equation and the predicted water contact angle by this model is depicted. It can be observed that the lowest F factor corresponds to the CA sample with the concentration of 15% w/v. This yields the highest predicted contact angle from Cassie’s model. This matches closely with the findings from the contact angle measurements which showed super hydrophilicity in the sample with the concentration of 15% w/v, when the other samples showed highly hydrophobic surfaces with contact angle range between 132° to 144°.

**Figure 5:**
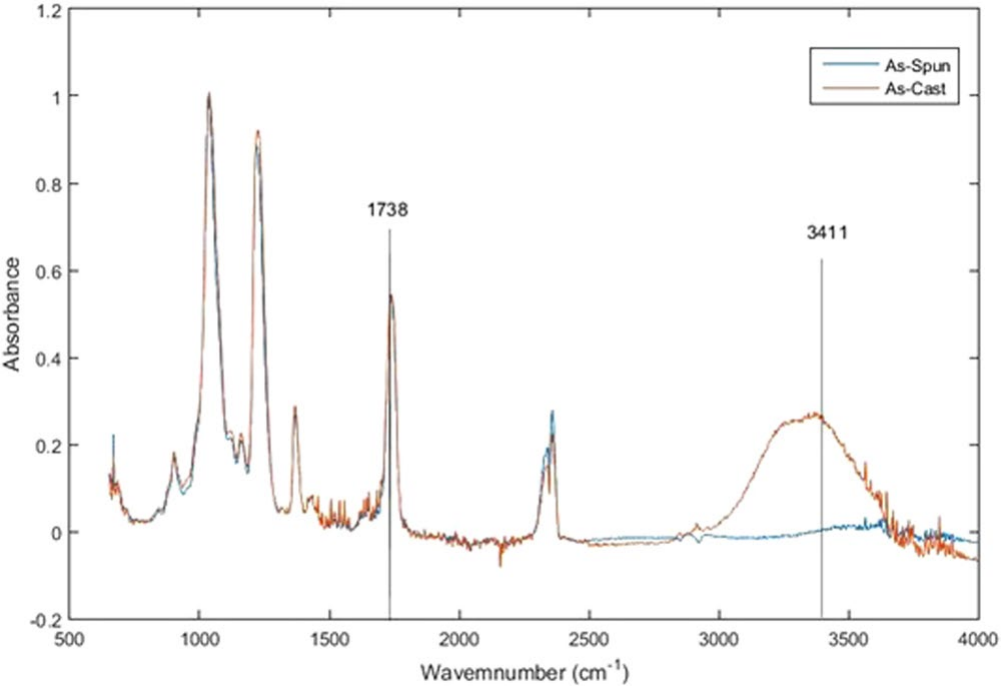
Normalized FTIR, Absorbance mode.

Studying the effect of the degree of substitution on hydrophobicity of CA^28^, Zhou *et al*. postulated that polarity of the surface in CA is determined by the presence of hydroxyl groups; thus by increasing the degree of substitution, which is a measure of the number of hydroxyl groups replaced by ester groups, the hydrophobicity of surface is increased. Based on this finding, the hydroxyl group of the as-spun CA vs as-cast CA is studied in this paper. Figure 5, draws a comparison between the normalized FTIR spectra of spin-coated CA film and as-spun CA mats. Prior to this test, both of the samples were dried in drying oven at 70 °c for 1 hour to eliminate the residual surface humidity of the fibers and the cast film. The three ester bands present in FTIR spectra of CA fibrous mats and spin-coated CA thin film at 1,740–1,745 cm^−1^, which are attributed to the symmetric stretching carbonyl group (C=O) of the acetyl group (−COCH3) in cellulose acetate^29^. The intensity of the band at 3400–3485 cm^−1^, represents the hydroxyl group (–OH)^30^. it can be observed that the intensity of the Hydroxyl band at 3450 cm^−1^ is decreased in as-spun fiber. Meaning that it might be possible that the concentration of the hydroxyl groups on the electrospun fiber is decreased during electrospinning process.

In order to compare the hydroxyl number of the as-spun and as-cast CA quantitatively, Beer-Lambert analysis on the FTIR spectrum was performed. FTIR spectrum used for this analysis was taken in Beer-lambert mod. [S2] The results are summarized in Table 1.

**Table 1:** Beer-Lambert analysis of the hydroxyl group.

Sample	Thickness (µm)	Absorbance 3411 (cm^−1^)	Absorbance Coefficient of Hydroxyl group (M^−1^cm^−1^)	Hydroxyl Concentration (M/cm^3^)
As- Spun	124	0.88	75	0.94
As-Cast	83	0.31	75	0.37

Decrease in hydroxyl groups drastically affects surface energy and fiber characteristics. This finding could address the reason behind the increase of hydrophobicity of the electrospun CA fibers compared to the CA films, but further studies on the effect of electrostatic field and polymer strechings on the hydroxyl groups is required.

**Figure 6:**
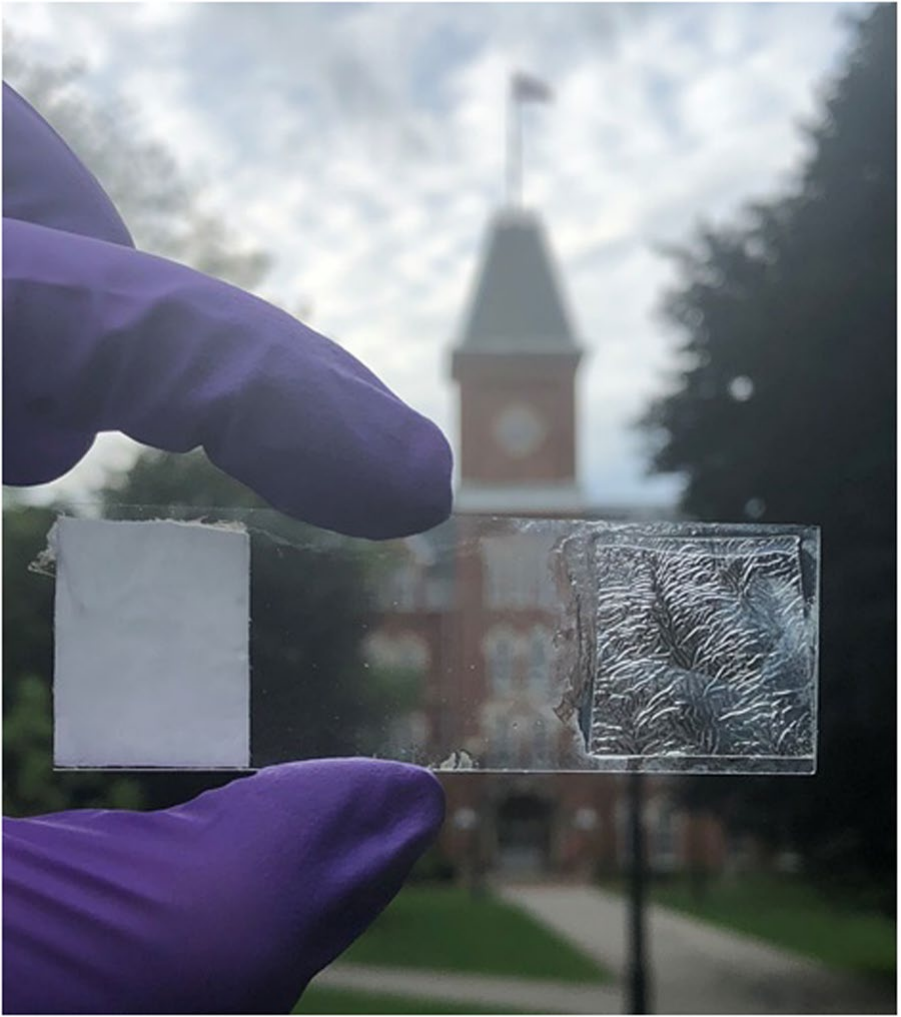
As-Spun CA on the left and As- Cast CA on the right.

As shown in Fig. 6, upon electrospinning of cellulose acetate mats, transparency changes drastically; Cellulose Acetate cast film being complete transparent and the electrospun fibers being totally opaque. This transformation gives a hint about a change in crystallinity and chain conformation of CA which is already pointed out in different studies^31–34^. It is possible that due to a change in crystallinity, intermolecular hydrogen was broken.

Previous studies on the water contact angle of the electrospun CA fibers by Shiratori *et al*., showed the water contact angle of CA after electrospinning was decreased to 0°, making it super hydrophilic^15,18^. Shiratori *et al*. argued that this change was related to the increase in the surface area of CA after electrospinning and proposed that the hydroxyl group on the surface had increased. However, these authors did not carry any surface measurements to quantify the number of hydroxyl bonds, nor did they provide any theoretical model for their arguments. In contrast, in the research described here, the relative change in the number of hydroxyl bonds has been measured both for the as-cast and the as-spun materials and the results fully support our argument.

**Figure 7:**
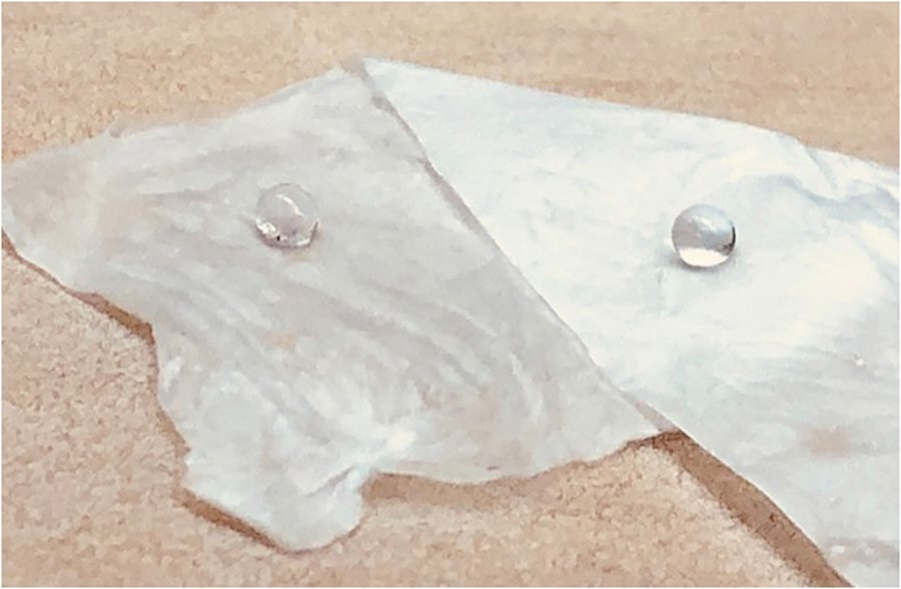
Water droplets on the two sides of the Electrospun CA fiber mat.

Even though there is a difference in the electrospinning precursor (molecular weight of the CA, the solvent used to prepare the precursor solution and the relative viscosity of the solution) and in the electrospinning process parameters (working distance and voltage) between the work presented here and in the other literature, which ultimately results in the final fiber to be completely different, these factors alone may not cause a contact angle shift from 0° to above 150^o^. It is possible that Shiratori *et al*. used the fibers that were not peeled off from the electrospinning collector or a surface which resulted in the observation of hydrophilic electrospun CA. In our work, we have observed that water droplets on as-spun CA fibers that have not been removed from the electrospinning collector plate absorb into the mat. This may have occurred because of the electrostatic force between the fibers and the grounded collector resulting from the residual charges present in the as-spun fibers. Therefore, making it impossible to measure the water contact angle without removing the effect of the external charge. The study presented here utilizes self-supported fibrous mats for contact angle measurements to eliminate any substrate or surface effects. After the fibers were peeled off from the collector, both sides of the mats were tested, and the results are shown in Fig. 7. It is apparent that there is no difference between any of the sides of the mats as it is removed from the grounded collector. Additionally, the Cellulose Acetate that was used in their study is different in the molecular weight. In electrospinning, using a polymer with higher molecular weight results in different viscosity, which ultimately changes the morphology, roughness and the outcome of the electrospun fibers.

Moreover, in another study on the hydrophobicity of cellulose triacetate (cellulose acetate with a higher degree of substitution), hydrophobicity was observed in the as-spun fibers^16^. This observation is consistent with the work reported here on CA. However, the observed effect was not explained and could not be investigated further. Additionally, since cellulose acetate with degree of substitution of 3.00 is used, it means that there were no hydroxyl groups present in the polymer chains; therefore, the reason and mechanism of the hydrophilic to hydrophobic change could not be isolated and tracked to the change in hydroxyl groups after electrospinning. The conclusion offered was that the concentration of the cellulose triacetate and the solvent ratio affected the water contact angle. On the other hand, this work provides a framework for processing super-water repellant CA mats in a single step electrospinning.

**Figure 8:**
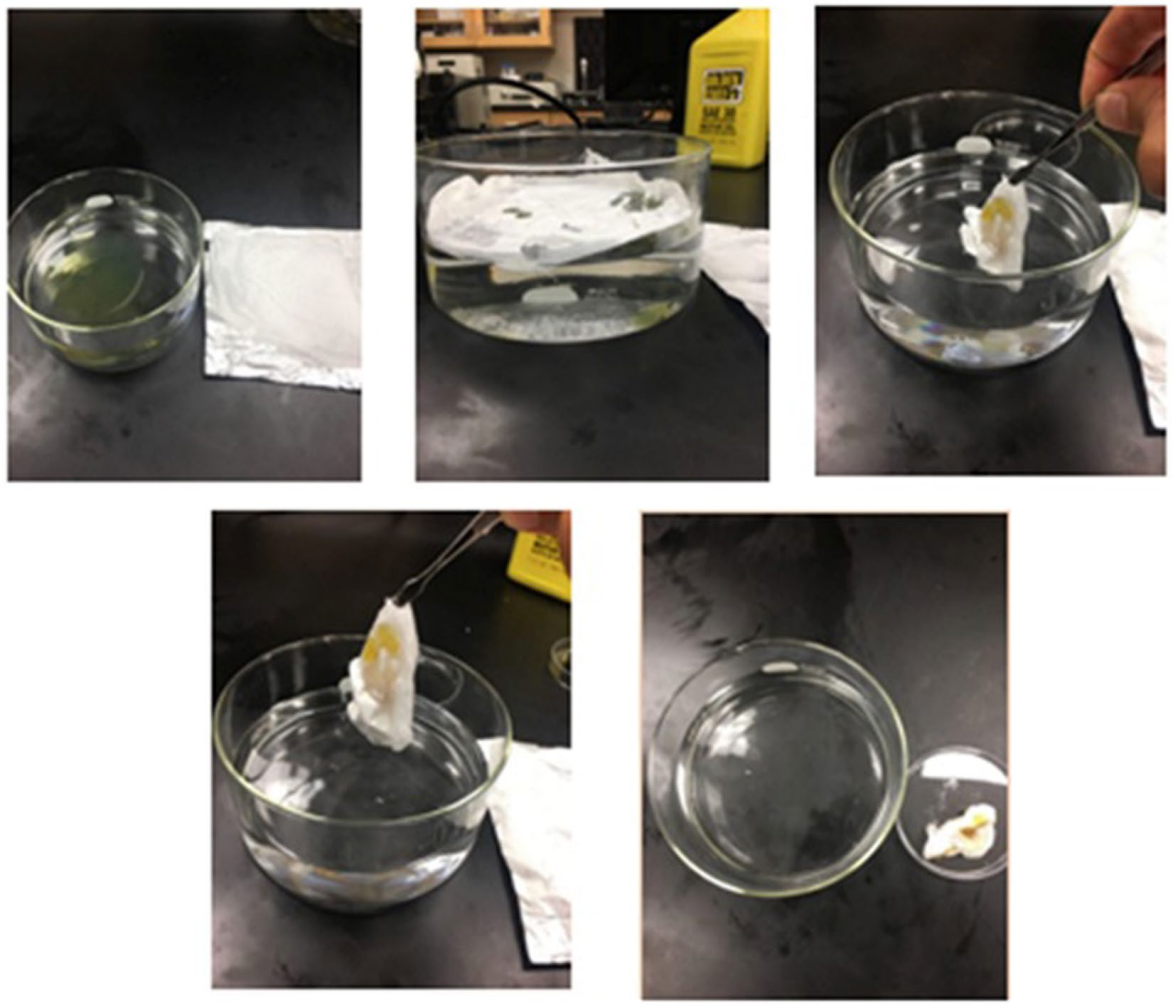
Testing the oil sorption efficiency of the CA electrospun mats.

Finally, the efficiency of the superhydrophobic mats as an oil spill removal sheets was assessed by adding 3 grams of motor oil to a petri dish containing tap water and 0.1 gram of electrospun CA was used to remove it. It was apparent that the fibers did not leave any oil contamination in the water but absorbed it all. The process of oil removal can be seen in Fig. 8.

## Conclusion

Cellulose Acetate electrospun mats were fabricated through the electrospinning process. During this process, a shift from a hydrophilic to a super water repellant surface was observed in one step without further surface modification and surface energy reduction. The as-spun fibers showed a water contact angle of 154.3 degrees classifying it as super-water repellant. Evidence from all of the techniques mentioned above confirms that while manipulating surface roughness through electrospinning increases the water contact angle, surface energy also reduces since the overal hydroxyl groups of Cellulose Acetate decreases after electrospinning. This work provides a framework for processing super-water repellant CA mats by single step electrospinning.

## Electronic supplementary material

Supplementary Information
