# Supplementary material for: Super Water-Repellent Cellulose Acetate Mats
Source: Sci Rep. 2018 Aug 20;8:12472. doi: 10.1038/s41598-018-30693-2 (PMC6102242; doi:10.1038/s41598-018-30693-2)
Supplement: Supplementary file 1 — Supplementary Information [file 41598_2018_30693_MOESM1_ESM.docx]

# Super Water-Repellent Cellulose Acetate Mats

# Fateh Mikaeili^1^, Pelagia I. Gouma^1,2*^

1. The Ohio State University, Department of Materials Science and Engineering
2. The Ohio State University, Department of Mechanical and Aerospace Engineering

Correspondence to: [Mikaeili.1@osu.edu](mailto:Mikaeili.1@osu.edu)

**Supplemental Data**

1. **Measurements of the predicted contact angle through Cassie’s Model**


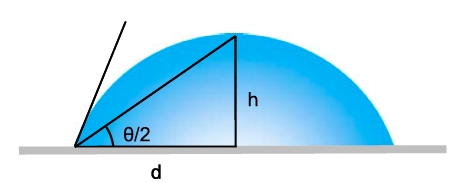
Basically, it can be understood from Cassie’s formula that by mesuring the apparent contact angle of a flat surface, the increase in the contact angle after reduing the solid/liquid point of contact, can be predicted..For predicting the apparent contact angle using Cassie’s model, it is necessary to obtain the F factor mentioned in the equation number one. F factor is the ratio of the solid in contact with the liquid to the whole area of the flat surface meaning that F factor for a flat surface should be 1.^14^ Therefore, calculating the area of the fibers in the zone that is covered by the water droplet, divided by the area that is covered by the water droplet should give us the F factor. On a flat sufrace, using drop shape analysis a simple equation can be derived to caclulate d which is radius of the projected area of liquid, and can be summerized in equation below:

$\frac{\theta}{2}={tan}^{-1}(\frac{h}{d}$)

Figure 5-Demonstration of Shape analysis using theta/2 method

Calculating d of the water droplet on the cast film of CA was mewsured to be 0.63 milimeters. Using the same area in the as-spun fibers, and calculating the area that is covered by the fibers would give us the area that water and fibers are in touch. Throguh image analysis and thresholding using a binary copy of the SEM image in MIPAR software, a rough estmiation of the area in which the fibers are present was calculated. The area of water solid contact in roughned surface is calcluated and then used in Cassies model in equation number 2.

1. **Real Images of Water droplet on as-spun fibers and Spin coated CA**


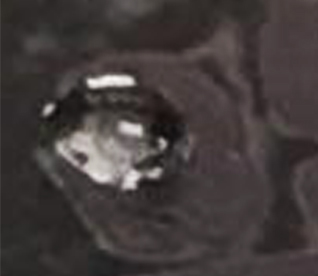

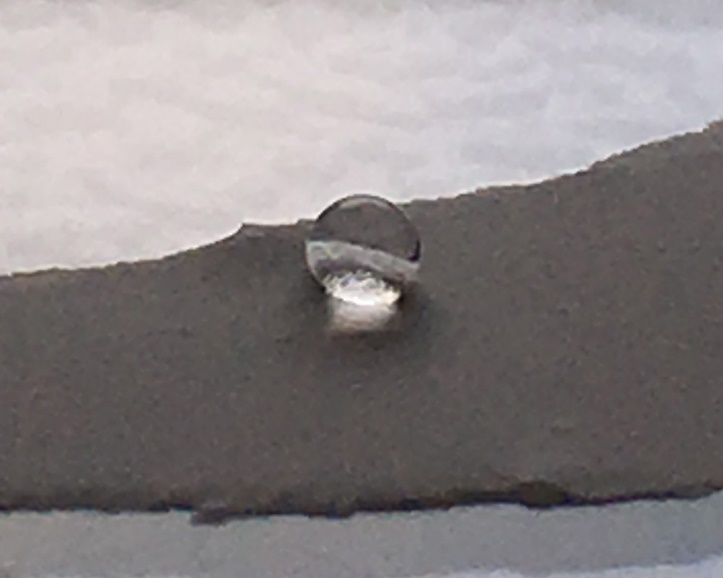
Figure 3 shows the actual images of water droplet interactions with each type of the CA surfaces of (a) electrospun CA fiber and (b) cast CA

Comparison between water droplet on (a) CA electrospun Mat (b) Casted CA film

1. **FTIR results in Beer-Lambert Mode
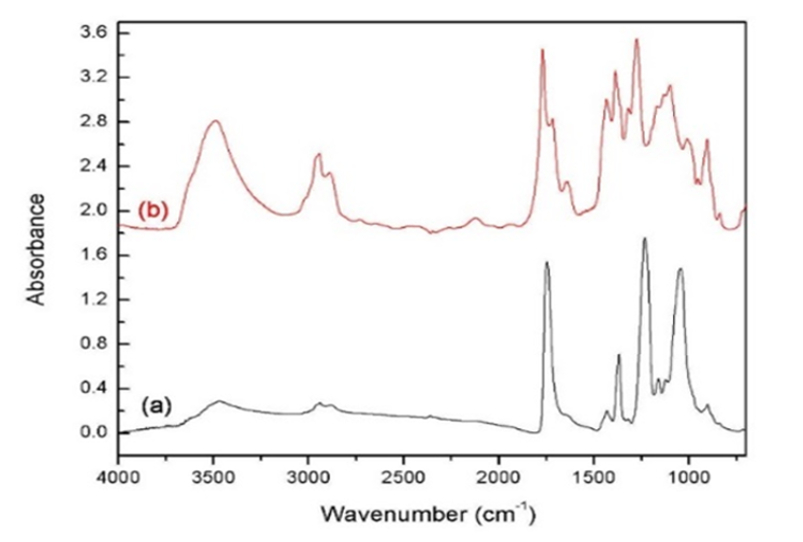
**
